# *Cambodian Beer Dreams*: The Consequences of Unchecked Alcohol Industry Power

**DOI:** 10.1177/14550725261502839

**Published:** 2026-11-12

**Authors:** Paula Rautoja, Sébastien Berret, Viivi Kauko, Eveliina Väkeväinen

**Affiliations:** 1 Faculty of Social Sciences, Centre for Research on Addiction, Control and Governance, University of Helsinki, Helsinki, Finland

*Cambodian Beer Dreams*, directed by Laurits Nansen. Denmark, Sweden, France: CAT&Docs, 2026, 82 min.

Screened at the 2026 Helsinki International Film Festival Rakkautta & Anarkiaa, Danish journalist Laurits Nansen's *Cambodian Beer Dreams* is an eye-opening documentary about the harms caused by the alcohol industry in a country where regulation has failed to keep pace with commercial interests. The documentary follows activist Kim Eng's struggle against “Big Alcohol” and its efforts to expand the beer market in a country that historically had a relatively limited drinking culture. It takes viewers into contemporary Cambodia, where alcohol advertising saturates public spaces and beer companies are deeply embedded in entertainment, politics and everyday life. Through neon-lit street parties, reality television sponsorships, as well as interviews with activists, shop owners and young women working as beer promoters, or ‘beer girls’, the documentary presents a disturbing picture of how multinational corporations exploit gaps in regulation.

*Cambodian Beer Dreams* offers valuable material for students, social scientists, and public health researchers. The film provides concrete examples of the commercial determinants of health: the social, political, and economic structures, as well as norms and practices, through which commercial actors affect population health (Freudenberg et al., 2021; Lacy-Nichols et al., 2023). Particular attention is given to harmful industry practices, especially alcohol marketing. While permeating public space, beer marketing in Cambodia is especially notable for its “gamblified” features. Macey and Hamari (2022) define *gamblification* as the “*(increased) presence of gambling (or gambling-related content) in non-gambling contexts in order to realize desired outcomes*”; for example, by using gambling games and gambling-like mechanisms. In Cambodia, where many people face economic hardship, beer companies offer goods and cash through promotional lotteries linked to beer-can tabs and bottle caps. In one unsettling scene in the film, a man drinks bottle after bottle in the hope of winning the jackpot. The scene illustrates how gamblification transforms the dream of sudden wealth into a powerful marketing tool that encourages continued alcohol consumption.

Another harmful industry practice depicted in the film concerns the treatment of the so-called ‘beer girls'. Employed in bars and nightclubs to promote beer and entice customers to drink more, these young women work under precarious conditions and are left to endure sexual harassment from customers with little protection. They also face pressure from employers who discourage them from joining unions that could defend their rights and improve their working conditions. The documentary also illustrates the *corporate playbook*: the strategies used by powerful commercial actors to protect their interests. Examples of alcohol industry lobbying and its consequences include beer companies donating cash and thousands of beer cases to Cambodian authorities, politicians holding shares in the alcohol industry, and the then prime minister publicly endorsing a beer brand. The film links this close relationship between political and commercial interests to the rejection of Kim Eng's proposed legislation to increase alcohol taxation.

Finally, Nansen's documentary offers a compelling illustration of the global political and economic drivers of ill health. Structural drivers such as neoliberalism, deregulation, and globalization have reshaped how commercial actors operate, while also influencing political and economic systems more broadly (Lacy-Nichols et al., 2023). Of these forces, globalization has arguably had the greatest impact on alcohol consumption in Cambodia. The film vividly portrays how multinational alcohol corporations from the Global North have expanded into the Global South and actively shaped local drinking cultures (Walls et al., 2020). It also draws a parallel between the erosion of Cambodia's fragile democracy and the growing influence of the alcohol industry. The increasing presence of European beer companies is presented alongside the country's political trajectory from early hopes of democracy towards authoritarian rule. *Cambodian Beer Dreams* thus offers a powerful case study of globalized neoliberal capitalism operating under weak regulation and with few effective constraints on commercial influence.

The developments depicted in the film are well documented in academic research. Although many countries in the Global South have lower levels of alcohol consumption than high-income countries, they experience a disproportionate burden of alcohol-related harm. Alcohol contributes to three million deaths worldwide each year (WHO, 2022), with the highest alcohol-attributable mortality rates found in low-income countries (WHO, 2024). While alcohol consumption in South-East Asia has historically been below the global average, it has increased rapidly over the past decade (Sornpaisarn et al., 2020). Cambodia, Vietnam, Lao PDR, Myanmar, and Timor-Leste are among the countries where consumption has risen most sharply, contributing to an increasing burden of alcohol-attributable harm (Shield et al., 2025).

*Cambodian Beer Dreams* is an urgent and disturbing film that exposes the human costs of an industry whose success depends on expanding consumption, often regardless of the consequences. It leaves viewers questioning both the ethical responsibilities of multinational corporations operating in vulnerable communities and the responsibility of governments to protect public health from powerful commercial interests.
